# Increased Temporal Lobe Beta Activity in Boys With Attention-Deficit Hyperactivity Disorder by LORETA Analysis

**DOI:** 10.3389/fnbeh.2020.00085

**Published:** 2020-06-30

**Authors:** Ching-Tai Chiang, Chen-Sen Ouyang, Rei-Cheng Yang, Rong-Ching Wu, Lung-Chang Lin

**Affiliations:** ^1^Department of Computer and Communication, National Pingtung University, Pingtung, Taiwan; ^2^Department of Information Engineering, I-Shou University, Kaohsiung, Taiwan; ^3^Departments of Pediatrics, Kaohsiung Medical University Hospital, Kaohsiung Medical University, Kaohsiung, Taiwan; ^4^Department of Electrical Engineering, I-Shou University, Kaohsiung, Taiwan; ^5^Department of Pediatrics, School of Medicine, College of Medicine, Kaohsiung Medical University, Kaohsiung, Taiwan

**Keywords:** attention-deficit hyperactivity disorder, low-resolution electromagnetic tomography, EEG, boys, beta band

## Abstract

**Aim:** Attention-deficit hyperactivity disorder (ADHD) is a common childhood neuropsychiatric disorder that affects 6.1 million US children. The mechanism of ADHD is currently unclear. Differences in ADHD presentations between boys and girls are well-established. In the present study, we used quantitative electroencephalography (EEG) to investigate the brain area and EEG bands of boys with ADHD.

**Methods:** This study enrolled 40 boys with ADHD and 40 age-matched controls without ADHD. Low-resolution electromagnetic tomography (LORETA) and instantaneous frequency were used to analyze EEG data to reveal the mechanisms underlying ADHD in boys.

**Results:** We found that the instantaneous frequencies in the T3 and T4 EEG channels in boys with ADHD were significantly higher than those in the controls. The beta band showed significant difference in current density between the ADHD and control groups. In the entire brain area, the bilateral inferior and middle temporal gyrus exhibited the most significant difference between the ADHD and control groups in the EEG beta band. Connectivity analysis revealed an increase in connectivity between the left middle frontal gyrus and fusiform gyrus of the temporal lobe in boys with ADHD.

**Conclusions:** LORETA is a promising tool for analyzing EEG signals and can be used to investigate the mechanism of ADHD. Our results reveal that the inferior temporal gyrus, middle temporal gyrus, and fusiform gyrus of the temporal lobe are potentially involved in the pathogenesis of ADHD in boys. In comparison with other imaging methods, such as magnetic resonance imaging, EEG is easy to perform, fast, and low cost. Our study presents a new approach for investigating the pathogenesis of ADHD in boys.

## Introduction

Attention-deficit hyperactivity disorder (ADHD) is the most common neurodevelopmental disorder diagnosed in childhood. An estimated 6.1 million US children 2–17 years of age (9.4%) have received an ADHD diagnosis (Danielson et al., 2018). In addition, ADHD is more commonly diagnosed in boys than in girls (the determined ratios range between 3:1 and 9:1) (Cormier, 2008; Willcutt, 2012). Sex differences in the phenotypic expression of ADHD are often used as an explanation for the greater rates of ADHD diagnosis in male individuals. Clinically, boys are more likely to have ADHD and score higher in all ADHD symptom domains compared with girls (Mowlem et al., 2019). Girls with ADHD are less hyperactive but are more inattentive and have higher risks of developing depression and anxiety disorders than do boys with ADHD (Gaub and Carlson, 1997; Quinn, 2005). Moreover, girls with ADHD more commonly exhibit high emotional reactivity and excessive talking (Quinn, 2005). These differences suggest diverse mechanisms for ADHD between the sexes. Several studies have reported possible mechanisms of ADHD, including environmental toxicity (Roberts et al., 2019), genetic factors (Hawi et al., 2015; Mogavero et al., 2018), epigenetic regulation (Tran and Miyake, 2017), neurotrophic factors (Tsai, 2017), and socioeconomic status (Russell et al., 2016). In related studies, a higher theta/beta ratio (TBR) in electroencephalography (EEG) bands was observed in the central area in patients with ADHD (Chabot and Serfontein, 1996; Arns et al., 2012). However, at least five more recent studies with both children and adults have not replicated this elevated TBR (Loo et al., 2009, 2013; Van Dongen-Boomsma et al., 2010; Ogrim et al., 2012; Liechti et al., 2013; Buyck and Wiersema, 2014). In addition, the exact mechanism of and brain area involved in ADHD in both sexes remain unclear. In our unpublished data, we found that brain maturation delay in the posterior brain areas might result in the inattention subtype of ADHD in girls. For a more complete understanding of the brain area and EEG bands involved in male individuals with ADHD, the present study examined a cohort of boys with ADHD.

The majority of ADHD studies have used magnetic resonance imaging (MRI) to investigate the mechanisms of ADHD (Noordermeer et al., 2017; Fernández-Jaén et al., 2018). However, MRI examination is expensive and time consuming. EEG is a non-invasive and convenient tool for studying brain function. Low-resolution electromagnetic tomography (LORETA) is an EEG source imaging technique that is frequently used to identify affected brain structures in patients with neurological diseases (Alonso et al., 2015), such as the seizure onset zone in patients with drug-resistant epilepsy (Staljanssens et al., 2017). In patients with moderate or severe obstructive sleep apnea, LORETA can be used to localize the generators of EEG activity in separate EEG frequency bands. Research revealed that EEG background activities were normalized by continuous positive airway pressure therapy in both moderate and severe obstructive sleep apnea groups (Toth et al., 2016). In ADHD, the pathogenetic brain area is unclear. To investigate the affected brain area and EEG bands in male individuals with ADHD, the present study used LORETA to analyze the EEG of a cohort of boys with ADHD.

## Materials and Methods

### Study Population

The study cohort comprised 40 boys with ADHD and 40 age-matched boys with no ADHD (controls). This study enrolled only boys to reduce the confounding effect of different sexes in ADHD. The mean age of patients in the ADHD group was 7 years, 7 months (±2 years, 0 months; ranging from 5 years, 1 month to 12 years, 8 months), and that of participants in the control group was 7 years, 11 months (±1 year, 4 months; ranging from 5 years, 2 months to 11 years, 6 months). No significant difference in age distribution was observed between the groups. All children underwent an examination and a detailed clinical interview with a pediatric neurologist or psychiatrist in addition to receiving an EEG evaluation. None of the boys were on any ADHD medication before the time of testing. Children with histories of epilepsy, intellectual disability, drug abuse, head injury, and psychotic disorders were excluded. ADHD was diagnosed according to *Diagnostic and Statistical Manual of Mental Disorders-V* (*DSM-V*) criteria and the Child Behavior Checklist (CBCL) scales, and the Swanson, Nolan and Pelham Teacher and Parent Rating Scale (SNAP-IV) was used to evaluate ADHD severity. The SNAP-IV consists of 26 items that are rated on a 4-point scale (“not at all,” “just a little,” “quite a bit,” and “very much”). The items are divided between three subscales: inattention (nine items), hyperactivity/impulsivity (nine items), and opposition (eight items). Subscale scores are calculated according to the average ratings. The items for inattention and hyperactivity/impulsivity can be combined to also create a “combined ADHD score” (Bussing et al., 2008). The diagnosis of ADHD should fulfill the core symptoms of the two domains (inattentive and hyperactive/impulsive) outlined in Table 1. Children with ADHD typically display six (or more) symptoms. All symptoms must be present in at least two settings and must clearly affect functioning (Cabral et al., 2020). Written informed consent was obtained from a family member or legal guardian of each child. This study was reviewed and approved by the Institutional Review Board of Kaohsiung Medical University Hospital [KMUIRB-SV(I)-20150052].

**Table 1:** Diagnostic features of ADHD (adapted from *DSM-V*).

**Hyperactivity and impulsivity**
Fidgets excessively
Cannot remain seated when required (e.g., in the classroom)
Feels restless
Cannot play quietly
Always “on the go” and seems to be “driven by a motor”
Talks excessively
Impatiently blurts out answers before questions are finished being asked
Cannot wait for his or her turn
Interrupts, intrudes, or takes over others' activities
**Inattention**
Fails to pay attention to details, makes careless mistakes
Cannot remain focused during work or play
Does not seem to listen when spoken to
Cannot follow instructions and fails to complete work
Cannot organize tasks and activities
Avoids tasks that require concentration, such as reviewing lengthy papers
Loses things needed for tasks and activities
Gets distracted by extraneous stimuli, such as unrelated thoughts
Forgetful in daily activities, such as paying bills and keeping appointments

*ADHD, attention-deficit hyperactivity disorder*.

### EEG Recordings

Identical equipment and procedures were used for EEG recordings for all patients. Patients with ADHD underwent EEG examinations for 20 min with eyes closed. Patients were tested in a quiet, air-conditioned room. All recordings were made during daylight hours (between 08:00 a.m. and 05:00 p.m.). EEG data were digitally obtained using 19 electrodes at a sampling rate of 256 Hz (EBNeuro Mizar 33, Florence, Italy). Amplifier characteristics were bandpass filtered between 0.5 and 60 Hz with 10,000 times gain, and electrodes were arranged according to the International 10–20 System.

### EEG Acquisition

The 80 boys enrolled in this study were divided into two groups: ADHD and control groups. For each boy, artifact-free EEG segments were acquired. Eighteen channels of monopolar montage were adopted for analysis. To ensure unbiased comparison, all EEG segments were acquired from artifact-free sections of EEG recordings conducted when participants were awake.

### EEG Instantaneous Frequency

The EEG electrodes were arranged according to the International 10–20 System. The positions of the 19 electrodes were Fp1, Fp2, F7, F3, Fz, F4, F8, T3, C3, Cz, C4, T4, T5, P3, Pz, P4, T6, O1, and O2. The reference electrode, namely Cz, was placed on the midline sagittal plane of the skull. To ensure unbiased comparisons, artifacts caused by eye and muscle movements were manually removed by an experienced neurologist after visual examination in the preprocessing step (Lin et al., 2019). For each participant, a 300 s artifact-free EEG signal was extracted for analysis. The recordings of photic and hyperventilation segments were excluded. Subsequently, EEG signals were normalized as *z* scores with zero mean and a standard deviation (SD) of 1 (Lin et al., 2019).

EEG signals are non-stationary (Nazarpour et al., 2007). The instantaneous frequency of a non-stationary signal is a time-varying parameter related to the average frequency present in the signal as it evolves (Boualem, 1992).

If *x*(*t*) is a real-time series measured from an EEG channel, its corresponding Hilbert transform signal can be calculated as follows:

(1) $$\begin{array}{l} \tilde{x}\left(t\right)\;=\;\frac{1}{\pi }PV\int_{-\infty }^{\infty }\frac{x\left(\tau \right)}{t-\tau }d\tau \end{array}$$

where *PV* represents the Cauchy principal value denoting the following operation (Chatterjee and Misra, 2015):

(2) $$\begin{array}{l} PV\int_{-\infty }^{\infty }\frac{x\left(\tau \right)}{t-\tau }d\tau =\underset{\varepsilon \to 0^{+}}{\text{lim}}\left[\int_{-\infty }^{t-\varepsilon }\frac{x\left(\tau \right)}{t-\tau }d\tau +\int_{t-\varepsilon }^{\infty }\frac{x\left(\tau \right)}{t-\tau }d\tau \right] \end{array}$$

Subsequently, *x*(*t*) and $\tilde{x}\left(t\right)$ are the real and imaginary parts, respectively, of an analytical signal:

(3) $$\begin{array}{l} y\text{(t)}=x\left(t\right)+j\tilde{x}\left(t\right)=a\text{(t)exp}\left[\text{j}\theta \text{(t)}\right] \end{array}$$

where the instantaneous amplitude of the analytical signal is

(4) $$\begin{array}{l} \text{a(t)}=\sqrt{{\left[\text{x(t)}\right]}^{2}+{\left[\tilde{\text{x}}\left(\text{t}\right)\right]}^{2}} \end{array}$$

and its instantaneous phase is

$$\begin{array}{l} \theta \text{(t)}=\tan^{-1}\frac{\tilde{x}\left(t\right)}{x\left(t\right)} \end{array}$$

After applying signal processing, Ville formulated a time–frequency distribution of the EEG signal, which is now commonly referred to as the Wigner–Ville distribution (WVD; Tagluk et al., 2005):

(5) $$\begin{array}{l} W\left(t,f\right)=\int_{-\infty }^{\infty }y\left(t+\frac{\tau }{2}\right)y^{*}\left(t-\frac{\tau }{2}\right)e^{-j2\pi f\tau }d\tau \end{array}$$

where the superscript ^*^ denotes the complex conjugate operation. The first moment of the WVD with respect to frequency yields the instantaneous frequency as follows:

(6) $$\begin{array}{l} f_{i}\left(t\right)=\frac{\int_{-\infty }^{\infty }fW\left(t,f\right)df}{\int_{-\infty }^{\infty }W\left(t,f\right)df} \end{array}$$

According to (6), we can calculate the instantaneous frequency of each participant in the ADHD and control groups (i.e., 40 individualized instantaneous frequencies were obtained for each group). A two-sample *t*-test was then performed to identify significant differences between the two group means for instantaneous frequency in each electrode.

### EEG Source Localization

To analyze the intracortical distribution of the electrical activity from the surface EEG data, LORETA employs a discrete, three-dimensionally distributed, linear weighted minimum norm inverse solution. Intracerebral volume was partitioned into 6,239 voxels with a spatial resolution of 5 mm. In total, 20 artifact-free 5-s EEG epochs were randomly selected from the 300 s recorded EEG for each participant. For statistical neuroimaging analysis of source current density, LORETA applies a statistical non-parametric mapping (SnPM) method (Holmes et al., 1996). On the basis of the transformed current density power, the difference in source localization of cortical oscillations was assessed using the voxel-by-voxel independent sample log F-ratio between groups for each frequency band. In the resulting statistical three-dimensional images, cortical voxels revealing significant differences were identified through a non-parametric randomization or permutation procedure for comparing the mean source power of each voxel and the distribution of the permuted values. In total, 5,000 data randomizations were used to determine the critical probability threshold values for the actually observed log F-ratio values with correction for multiple comparisons across all voxels and all frequencies (Canuet et al., 2012).

### EEG Functional Connectivity

These measures regarding functional connectivity are defined in the frequency domain and are applicable to non-stationary EEG signals. A total of 20 artifact-free 5-s EEG epochs were randomly selected from the 300 s recorded EEG for each participant. A voxel-wise approach using regions of interest (ROIs) was applied for functional connectivity analysis. With emphasis on the temporal lobe, 70 seeds located around the entire cortical area were selected to compare the functional connectivity between the two groups (Yeo et al., 2011). The single nearest voxel was selected for defining the ROIs from the 70 seed points (Table 2). Total non-linear connectivity, defined as the sum of instantaneous non-linear connectivity and lagged non-linear connectivity, was used as a measure of functional connectivity between all pairs of ROIs (Pereda et al., 2005; Pascual-Marqui, 2007).

**Table 2:** Location of seed regions usedx for analysis of EEG functional connectivity.

**Seed Regions**	**Coordinates**			**Brodmann area**
**Frontal Lobe**				
Medial frontal gyrus	±5	50	30	9
Cingulate gyrus	±10	10	40	32
Middle frontal gyrus	±25	−5	50	6
Middle frontal gyrus	±30	40	30	9
Inferior frontal gyrus	±40	35	15	46
Inferior frontal gyrus	±40	55	5	10
**Limbic Lobe**				
Cingulate gyrus	±15	−30	40	31
Cingulate gyrus	±5	0	30	24
Cingulate gyrus	±5	20	45	32
**Parietal Lobe**				
Inferior parietal lobule	±60	−35	40	40
Angular gyrus	±50	−70	30	39
Inferior parietal lobule	±40	−35	40	40
Inferior parietal lobule	±50	−50	50	40
**Temporal Lobe**				
Middle temporal gyrus	±55	−10	−20	21
Fusiform gyrus	±55	−20	−30	20
Fusiform gyrus	±55	−40	−30	20
Fusiform gyrus	±55	−50	−25	37
Fusiform gyrus	±60	−10	−30	20
Fusiform gyrus	±60	−15	−30	20
Inferior temporal gyrus	±55	−10	−35	20
Inferior temporal gyrus	±60	−30	−25	20
Inferior temporal gyrus	±60	−40	−20	20
Inferior temporal gyrus	±65	−10	−20	21
Inferior temporal gyrus	±65	−20	−20	20
Inferior temporal gyrus	±65	−30	−20	20
Middle temporal gyrus	±60	−30	−10	21
Middle temporal gyrus	±65	−10	−10	21
Middle temporal gyrus	±65	−20	−10	21
Middle temporal gyrus	±65	−40	−10	21
Superior temporal gyrus	±55	−25	0	22
Superior temporal gyrus	±65	−10	0	21
Superior temporal gyrus	±65	−20	0	22
**Occipital Lobe**				
Cuneus	±10	−90	35	19
Cuneus	±10	−80	35	19
Cuneus	±5	−85	35	19

To assess the difference in total non-linear connectivity between the pairs of 70 ROIs ($C_{2}^{70}=\;$2,415 connections) in each frequency band for the ADHD vs. control groups, LORETA was used to perform independent sample *t*-tests to obtain *t* statistic images of EEG functional connectivity. For each analysis, 19,320 tests were performed using LORETA to compare all connections between 70 ROIs for δ, θ, α_1_, α_2_, β_1_, β_2_, β_3_, and total bands (2, 415 × 8 = 19, 320). Moreover, the LORETA non-parametric randomization procedure based on the “maximal statistic” was used to correct for multiple comparisons (Holmes et al., 1996). The omnibus null hypothesis was rejected if at least one *t* value (i.e., voxel t_*max*_) was above the critical threshold t_*crit*_ for *p* = 0.05, as determined through 5,000 data randomizations (Canuet et al., 2012).

### Statistical Analysis

All statistical analyses for instantaneous frequency were conducted using MATLAB software. Data are presented as the mean ± SD. A comparison of ADHD and non-ADHD EEG features was conducted using a two-sample *t*-test, where *p* < 0.05 was considered statistically significant. The SnPM method was applied in LORETA to analyze source current density in cortical voxels. The difference in source localization of cortical oscillations between the two groups for each frequency band was assessed using voxel-by-voxel independent sample F-ratio tests on the basis of log-transformed current density power. LORETA was used with 5,000 data randomizations to determine the critical probability threshold values for the actually observed log F-ratio values with correction for multiple comparisons across all voxels and all frequencies. In the resulting statistical three-dimensional images with a threshold at the 5% probability level, cortical voxels revealing significant differences were identified through a non-parametric randomization or permutation procedure for comparing the mean source power of each voxel and the distribution of the permuted values (Holmes et al., 1996). EEG connectivity was accessed for each frequency band by conducting independent sample *t*-tests to obtain three-dimensional images of brain connectivity; *p*-values were corrected for the multiple comparison tests by using the non-parametric randomization procedure available in LORETA. For each analysis, 19,320 tests were performed to compare 2,415 connections between 70 ROIs for each of the four frequency bands using 5,000 data randomizations.

## Results

The SNAP scores of patients with ADHD obtained from parents and teachers were 60.550 ± 7.438 and 41.306 ± 19.125, respectively. We further divided the SNAP scores into ADHD and oppositional scores. The ADHD scores of SNAP-IV from the parents and teachers were 44.054 ± 5.939 and 32.686 ± 12.755, respectively, and the oppositional scores were 16.496 ± 4.529 and 8.620 ± 7.358, respectively. In the control group, the SNAP score obtained from the parents was 11.425 ± 6.365. The SNAP scores were significantly higher in the ADHD group compared with the control group (Table 3). Regarding the CBCL scales, T scores from the parents and teachers were 72.971 ± 5.107 and 66.515 ± 9.558, respectively (Table 3). According to Achenbach, T scores over 63 represent the clinically significant range (Achenbach, 1991).

**Table 3:** Demographic data of boys with ADHD and controls.

	**ADHD** **(*N* = 40)**	**Control** **(*N* = 40)**	***p*-value**
Age	7 y 7 m ± 2 y 0 m	7 y 11 m ± 1 y 4 m	0.215
SNAP (parents)	60.550 ± 7.438	11.425 ± 6.365	<0.001
SNAP (teachers)	41.306 ± 19.125	NA	NA
T scores of CBCL (parents)	72.971 ± 5.107	NA	NA
T scores of CBCL (teacher)	66.515 ± 9.558	NA	NA

*NA, not available*.

### Comparison of Instantaneous Frequency and Current Density Between ADHD and Control Groups

**Figure 1:**
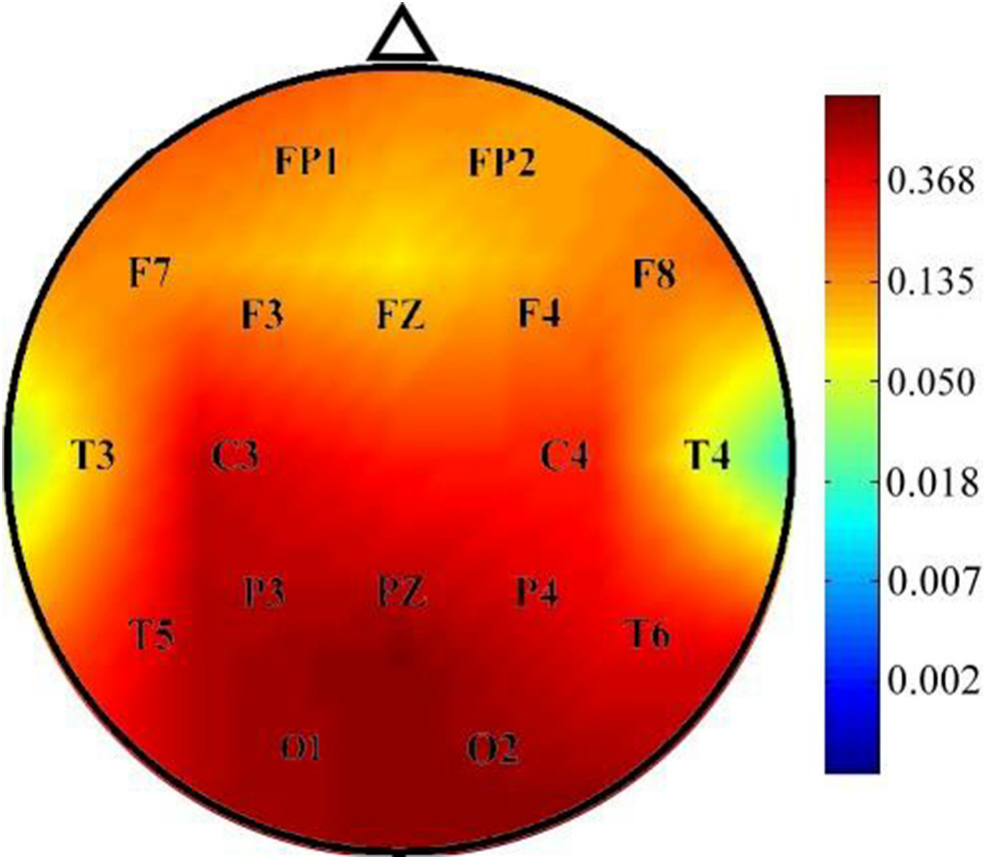
Comparison of *p*-values of instantaneous frequency between the ADHD and control groups. Instantaneous frequency values were significantly higher in the ADHD group than in the control group over the T3 and T4 channels.

**Figure 2:**
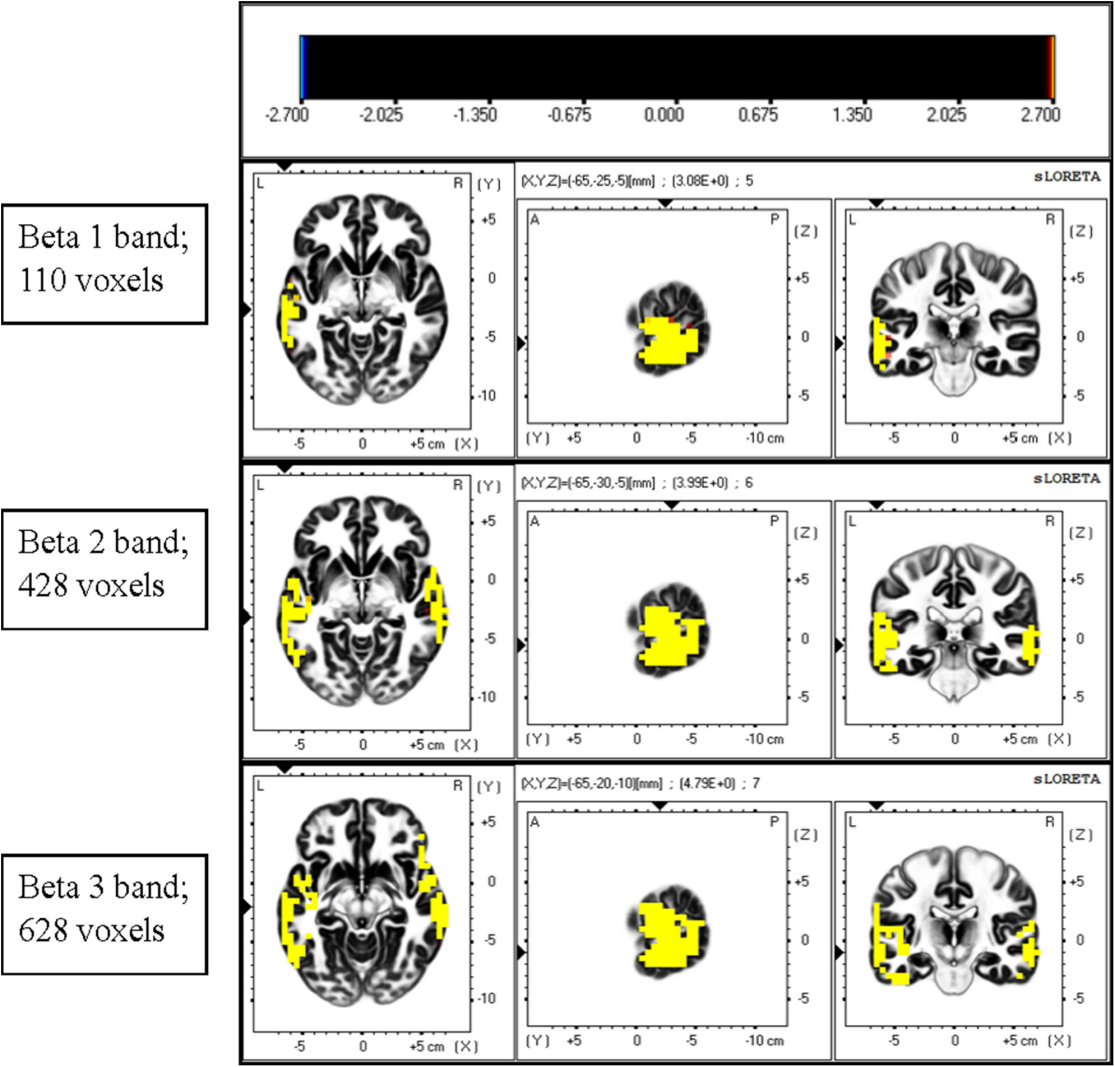
Comparison of current density of different EEG bands between the ADHD and control groups. Current density was significantly higher in the beta band in the ADHD group than in the control group. A significant difference was observed between the ADHD and control groups for the highest number of voxels in the beta 3 band. The most significant differences were observed over the inferior temporal gyrus and middle temporal gyrus.

The instantaneous frequencies in lateral temporal EEG channels in the ADHD group were higher than those in the control group. The differences were significant in the T3 (*p* = 0.027) and T4 (*p* = 0.013) channels (Table 4, Figure 1). EEG signals were further divided into different frequency bands: delta (1.5–4.0 Hz), theta (4.0–8.0 Hz), alpha 1 (8.0–10.0 Hz), alpha 2 (10.0–12.0 Hz), beta 1 (12.5–18.0 Hz), beta 2 (18.5–21.0 Hz), and beta 3 (21.5–30.0 Hz). The beta 1, 2, and 3 EEG bands exhibited a significantly higher current density in the ADHD group compared with the control group, including inferior temporal gyrus, middle temporal gyrus, superior temporal gyrus, precentral gyrus, postcentral gyrus, middle occipital gyrus, insula, fusiform gyrus, and inferior frontal gyrus (log F = 2.747, *p* < 0.05). In the entire brain area, the inferior temporal gyrus (Brodmann area 20) and middle temporal gyrus (Brodmann area 21) exhibited the largest difference between the ADHD and control groups (Figure 2).

**Table 4:** Comparisons of instantaneous frequency between the ADHD and control groups.

**EEG channel**	**ADHD (Hz)**	**Control (Hz)**	***p*-value**
FP1	7.316 ± 0.683	7.138 ± 0.696	0.258
FP2	7.290 ± 0.697	7.059 ± 0.601	0.121
F7	7.358 ± 0.737	7.141 ± 0.641	0.170
F3	7.574 ± 0.771	7.308 ± 0.756	0.128
Fz	7.114 ± 0.692	6.829 ± 0.718	0.078
F4	7.682 ± 0.880	7.397 ± 0.813	0.142
F8	7.304 ± 0.686	7.122 ± 0.632	0.227
T3	8.038 ± 1.185	7.555 ± 0.637	0.027^*^
C3	7.961 ± 0.834	7.847 ± 0.776	0.534
C4	7.973 ± 0.858	7.782 ± 0.775	0.304
T4	8.126 ± 1.262	7.541 ± 0.694	0.013^*^
T5	7.764 ± 0.829	7.520 ± 0.650	0.152
P3	7.714 ± 0.837	7.628 ± 0.729	0.630
Pz	7.475 ± 0.726	7.438 ± 0.624	0.810
P4	7.693 ± 0.856	7.567 ± 0.620	0.457
T6	7.624 ± 0.830	7.510 ± 0.598	0.488
O1	7.709 ± 0.796	7.631 ± 0.707	0.649
O2	7.615 ± 0.812	7.637 ± 0.632	0.891

* *p < 0.05*.

### Comparison of Functional Connectivity Between ADHD and Control Groups

**Figure 3:**
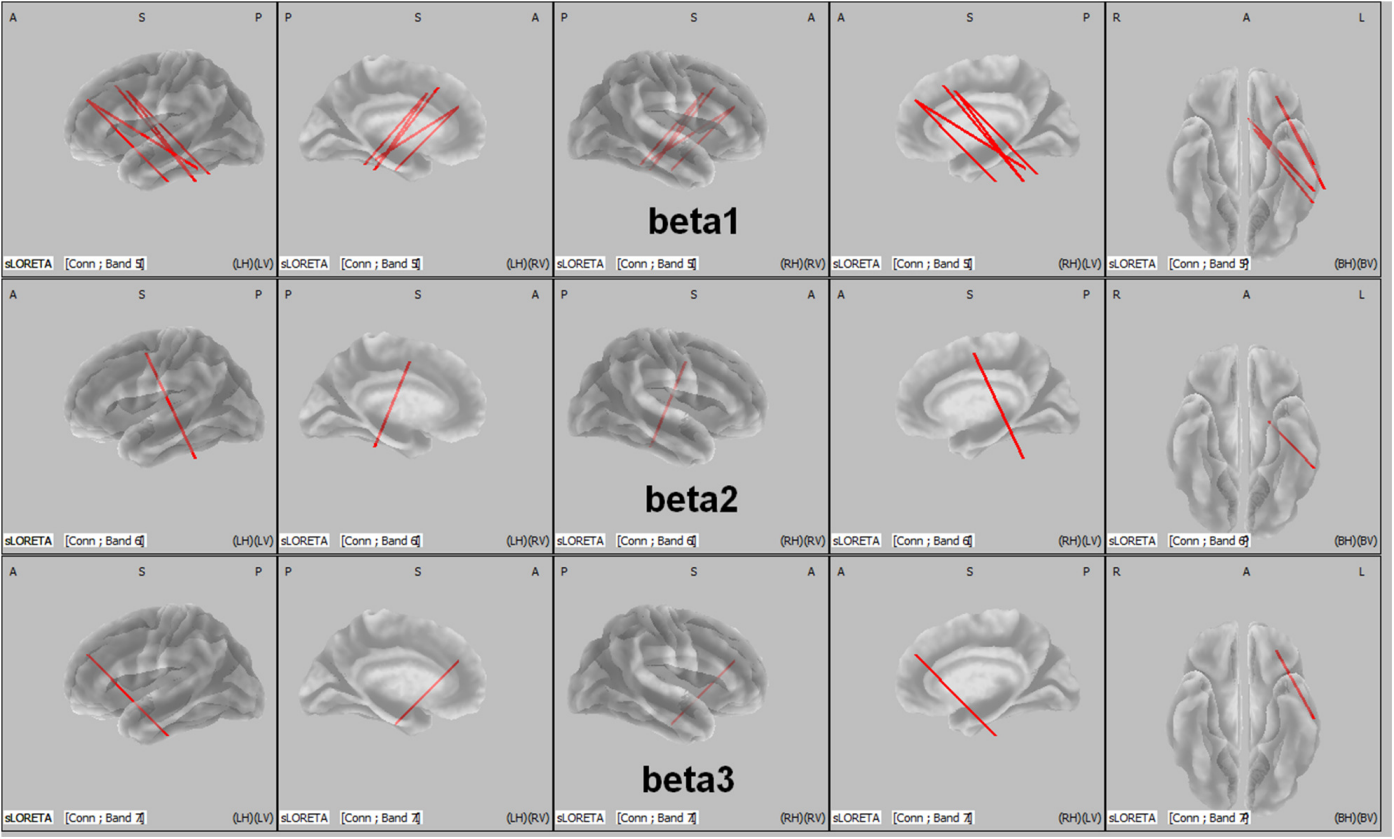
LORETA wire diagram. Cortical areas with significantly increased total non-linear connectivity of different beta bands for ADHD vs. controls. The greatest difference was observed in the connection between the middle frontal gyrus and the inferior temporal gyrus (threshold: *t* = 4.5810, *p* < 0.05). L, left; R, right; A, anterior; P, posterior.

The total non-linear connectivity in all brain areas and EEG bands was compared. The results indicated that the connectivity of all EEG bands over the left frontal area in patients with ADHD was significantly higher than that in controls (t = 4.5810, *p* < 0.05, corrected; Figure 3). The maximum *t* value and corresponding connected seed regions were listed in Table 5. In comparisons between the two groups, the greatest differences were observed in the connection between the left middle frontal gyrus and the fusiform gyrus of the temporal lobe for the beta 1, beta 2, and beta 3 bands with *p-*values of 0.031, 0.0422, and 0.0480, respectively. This discrepancy indicated that connectivity disturbance also plays a potential role in the pathogenesis of ADHD.

**Table 5:** Maximum *t* values of seed regions with a significant difference in functional connectivity between the ADHD and control groups.

**Frequency bands**	***t*-value (maximum)**	***p*-value**	**Seed regions (BA)/coordinates**	
β_1_	4.9316	0.0310	Middle frontal gyrus (9) (−30 40 30)	Fusiform gyrus (20) (−55 −20 −30)
β_2_	4.8511	0.0422	Middle frontal gyrus (6) (−25 −5 50)	Fusiform gyrus (20) (−55 −40 −30)
β_3_	4.8111	0.0480	Middle frontal gyrus (9) (−30 40 30)	Fusiform gyrus (20) (−55 −20 −30)

## Discussion

In this study, we found that the instantaneous frequencies in the T3 and T4 EEG channels in boys with ADHD were significantly higher than those in boys without ADHD. Through LORETA analysis, the differences in current density between the two groups were most prominent in the beta band over the inferior temporal gyrus and the middle temporal gyrus. In addition, the connectivity disturbance was significant between the left middle frontal gyrus and the fusiform gyrus of the temporal lobe for the beta 1, beta 2, and beta 3 bands in ADHD boys.

Several studies have reported that elevated theta activity, reduced alpha and beta activity, and elevated theta/alpha ratio and TBR are the most consistent hallmarks of ADHD (Barry et al., 2003; Hermens et al., 2005; Newson and Thiagarajan, 2018). However, recent studies have not reported any elevation of theta activity or TBR in ADHD. Our study revealed an increased current density of beta bands in boys with ADHD compared with that in the controls. Only a few studies have demonstrated elevated beta power in the EEG signals of patients with ADHD. For example, Lee et al. assessed the relationship between high-frequency EEG power, subjective inattention symptoms, adult ADHD symptoms, and childhood traumatic experience in 157 healthy adult volunteers. The participants were divided into two groups according to their Childhood Traumatic Questionnaire (CTQ) scores. The researchers found that the high CTQ group exhibited significantly increased beta 1, beta 2, and beta 3 band power, which significantly correlated with their inattention scores. Furthermore, the inattention scores significantly correlated with frontal beta 1, frontal beta 2, centrotemporal beta 1, and global beta 1 band power (Lee et al., 2017). In addition, Ogrim et al. investigated the EEG spectra and behavior data of 62 children with ADHD in comparison with those of 39 age- and sex-matched controls. The results demonstrated a positive correlation between absolute beta band power and inattention score in children and adolescents with ADHD and a negative correlation between beta band power and omission errors in control individuals (Ogrim et al., 2012). These results support the assumption that beta activity represents attention level. The discrepancy in EEG findings between these studies could be attributed to the heterogeneous characteristics of the included study groups. In future studies, sex, age, type of ADHD, and other comorbid diseases should be controlled to address this discrepancy.

ADHD is a heterogenous disorder that may be associated with other comorbidities, such as autism spectrum disorder, oppositional defiant or conduct disorder, intellectual disability, personality disorders, schizophrenia, and substance use disorders (Ottosen et al., 2019). According to previous reports, this disorder may involve multiple brain areas (consistent with the highly variable behavioral problems associated with this disorder), such as reduced total gray matter volume and reduced volume of the frontal cortex, basal ganglia, and the cerebellum (Noordermeer et al., 2017). In this study, we noted a significantly increased current density of the beta bands over the inferior temporal gyrus and middle temporal gyrus in boys with ADHD. These two areas are believed to play a major role in recognition memory, visual processing, auditory processing, and language. Choi et al. assessed the effects of aerobic exercise in 35 adolescents with ADHD undergoing methylphenidate treatment (Choi et al., 2015). Those engaging in exercise for 6 weeks exhibited significantly lower ADHD rating scale values than did their counterparts without exercise. The mean beta value on the functional MRI for the right temporal lobe in the exercise group decreased; however, no corresponding change was observed in the control group (Choi et al., 2015). This indicates that the temporal lobe may mediate attention processing. The temporal lobe, as a part of the limbic system (amygdala and hippocampus), is thought to play a role in the process of focusing on a task and acting quickly in the presence of distracting stimuli (Sterzer et al., 2005). In a meta-analysis, children with ADHD exhibited some hypoactivated brain areas and some hyperactivated brain areas compared with children without ADHD (Cortese et al., 2012). Moreover, ADHD-related hypoactivation is predominantly observed within the ventral attention and frontoparietal networks, whereas ADHD-related hyperactivation was predominantly observed within the default, ventral attention and somatomotor networks (Cortese et al., 2012). The default network comprises the inferior temporal gyrus, which showed increased beta band activity in the boys with ADHD in our study. In accordance with the aforementioned studies, our results suggest a possible association between elevated beta activity over the temporal lobe and ADHD symptoms in our study population.

So far, very few studies have used LORETA to explore the mechanisms of ADHD through EEG analysis. In contrast to imaging approaches such as MRI, EEG is a non-invasive, low-cost, fast, and convenient tool for studying brain function. In summary, using the promising analytic tool of LORETA, our study revealed that increased beta activity over the inferior temporal gyrus and the middle temporal gyrus and connectivity disturbance between the left middle frontal gyrus and the fusiform gyrus of the temporal lobe are potentially involved in ADHD manifestations in boys.

This study had some limitations. First, the sample size was insufficient to account for heterogeneity among the patients with ADHD. Second, the age distribution was wide in our participants with ADHD, ranging from 5 years, 1 month to 12 years, 8 months. The large age range may have influenced the results of EEG analysis. Future studies that control for age and type of ADHD and enroll more participants are required to broaden our findings.

## Conclusion

The mechanism of ADHD is unclear. LORETA is a promising tool for analyzing EEG signals and can be used to investigate the mechanism of ADHD. Our results revealed that the inferior temporal gyrus and the middle temporal gyrus might be involved in the pathogenesis of ADHD in boys. Elevated beta activity over the temporal area might have been associated with their ADHD symptoms. In addition, connectivity disturbance between the left middle frontal gyrus and the fusiform gyrus of the temporal lobe was observed in the boys with ADHD. Instead of other imaging methods such as MRI, EEG is easy to perform, fast, and low-cost. Our study presents a new approach for investigating the pathogenesis of ADHD in boys. Furthermore, this method could be extended to explore the brain areas involved in other neurological diseases.

## Data Availability Statement

The datasets generated for this study are available on request to the corresponding author.

## Ethics Statement

This study was reviewed and approved by the Institutional Review Board of Kaohsiung Medical University Hospital [KMUIRBSV(I)-20150052]. Written informed consent to participate in this study was provided by the participants' legal guardian/next of kin.

## Author Contributions

C-TC contributed to the study conception and design, contributed to acquisition and interpretation, and drafted and critically revised the manuscript. C-SO and R-CY contributed to the study conception and design, contributed to analysis and interpretation, and critically revised the manuscript. R-CW contributed to the study conception and design, contributed to acquisition and interpretation, and critically revised the manuscript. L-CL contributed to the study conception and design, contributed to analysis and interpretation, drafted and critically revised the manuscript, and provided final approval. All authors accept accountability for all aspects of the work and ensuring its integrity and accuracy.

## Conflict of Interest

The authors declare that the research was conducted in the absence of any commercial or financial relationships that could be construed as a potential conflict of interest.
